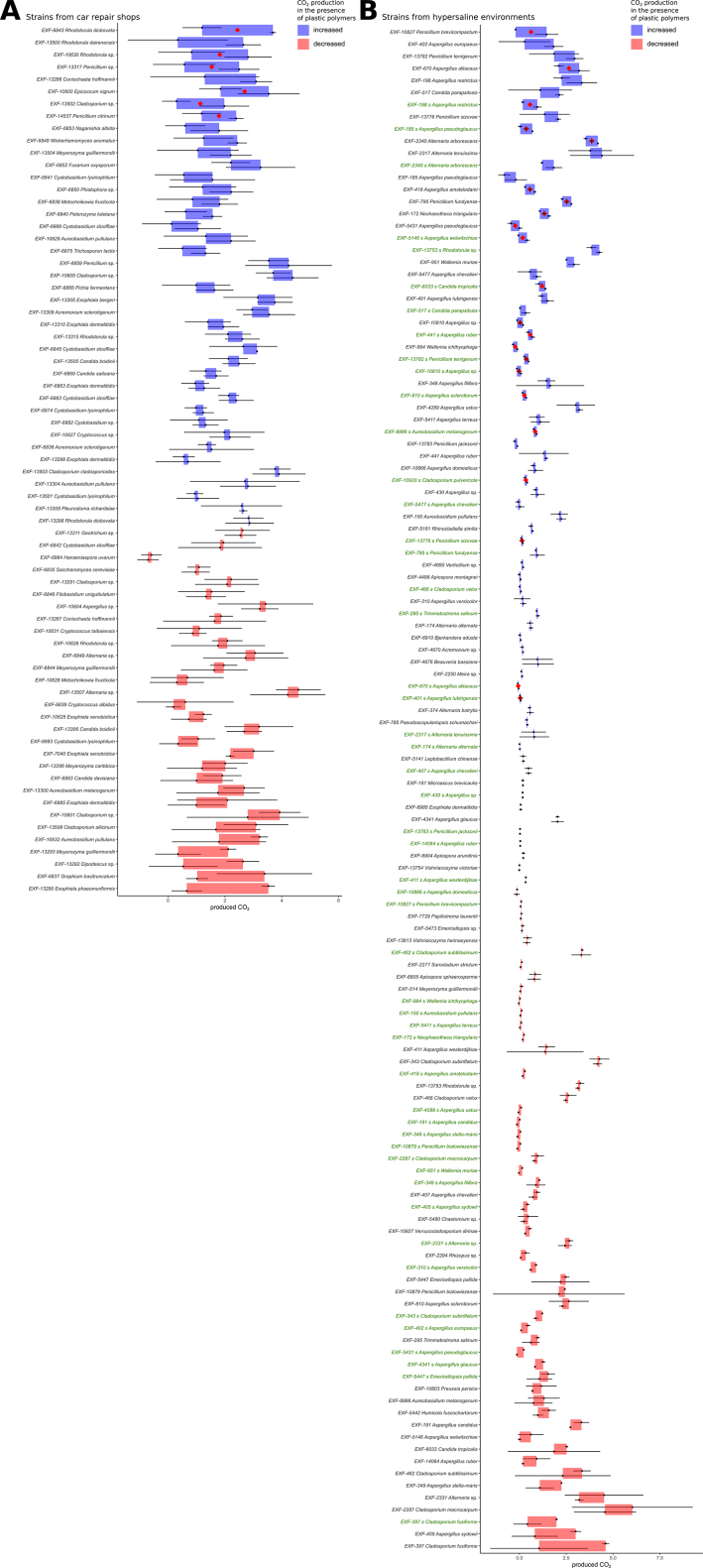# A screening method for plastic-degrading fungi

**DOI:** 10.1016/j.heliyon.2024.e31130

**Published:** 2024-05-11

**Authors:** Anja Černoša, Antonio Martínez Cortizas, Mohamed Traoré, Matejka Podlogar, Tjaša Danevčič, Nina Gunde-Cimerman, Cene Gostinčar

**Affiliations:** a University of Ljubljana, Biotechnical Faculty, Department of Biology, Jamnikarjeva 101, 1000 Ljubljana, Slovenia; b InnoRenew CoE, Livade 6a, 6310 Izola, Slovenia; c CRETUS, EcoPast research group (GI-1553), Departamento de Edafoloxía e Química Agrícola, Faculty of Biology, Universidade de Santiago de Compostela, Campus Vida, 15782, Spain; d Bolin Centre for Climate Research, Stockholm University, Stockholm, Sweden; e Department for Nanostructured Materials, Jožef Stefan Institute, Jamova cesta 39, Ljubljana, Slovenia; f University of Ljubljana, Biotechnical Faculty, Department of Microbiology, Jamnikarjeva 101, 1000 Ljubljana, Slovenia

**Keywords:** Screening, Method, Plastic degradation, Biodegradation, Fungi, Gas chromatography, SEM, FTIR, Raman spectroscopy

## Abstract

The growing amount of plastic waste requires new ways of disposal or recycling. Research into the biodegradation of recalcitrant plastic polymers is gathering pace. Despite some progress, these efforts have not yet led to technologically and economically viable applications. In this study, we show that respirometric screening of environmental fungal isolates in combination with scanning electron microscopy (SEM), Fourier transform infrared spectroscopy (FTIR) and Raman spectroscopy can be used to identify new strains with the potential for the degradation of plastic polymers. We screened 146 fungal strains, 71 isolated from car repair shops, an environment rich in long-chain hydrocarbons, and 75 isolated from hypersaline water capable of growing at high concentrations of NaCl. When grown in a minimal medium with no carbon source, some strains produced significantly more CO_2_ when a pure plastic polymer was added to the medium, some only at high salinity. A selection of these strains was shown by FTIR and Raman spectroscopy to alter the properties of plastic polymers: *Cladosporium* sp. EXF-13502 on polyamide, *Rhodotorula dairenensis* EXF-13500 on polypropylene, *Rhodotorula* sp. EXF-10630 on low-density polyethylene and *Wickerhamomyces anomalus* EXF-6848 on polyethylene terephthalate. Respirometry in combination with specific spectroscopic methods is an efficient method for screening microorganisms capable of at least partial plastic degradation and can be used to expand the repertoire of potential plastic degraders. This is of particular importance as our results also show that individual strains are only active against certain polymers and under certain conditions. Therefore, efficient biodegradation of plastics is likely to depend on a collection of specialized microorganisms rather than a single universal plastic degrader.

## 1 Introduction

Plastic pollution is one of the biggest environmental problems of our time. Improper disposal, inadequate recycling and the shortcomings of existing methods of managing plastic waste have led to the accumulation of plastic polymers in the environment. While plastic items fragment over time into ubiquitous micro- and nanoplastics, the hazards of which are only beginning to be understood [1], depolymerisation and mineralisation of plastics is exceptionally slow [2]. Even for properly collected plastic waste, the existing processing options are limited and have significant shortcomings. Recycling is complicated by the many different types and combinations of plastic polymers and additives. Incineration produces CO_2_ and, if not properly managed, toxic emissions. As a result, 46 % of the world's plastic waste is still sent to landfill, 22 % is mismanaged or not collected at all, 17 % is incinerated, and only 15 % is collected for recycling [3].

Plastic pollution will remain a problem for the foreseeable future. Despite legislative initiatives such as the ban on single-use plastic products in the European Union and the development of biodegradable plastics, the production of conventional, non-biodegradable plastics continues to increase [4]. This increases the need for better and more diverse methods of plastics management [5]. One of the promising approaches is biodegradation by microorganisms or their enzymes [2,6,7].

Plastics are synthetic polymers, usually derived from fossil fuels, consisting of long chains of repeating monomers. Their durability, low cost, and versatility have led to their use in numerous applications, from packaging materials to building components [8]. However, part of their durability is due to their poor biodegradability, which stems from several factors. Long polymer chains are densely packed and hydrophobic, often crystalline, they are often mixed with antimicrobial additives, and they are unlike natural polymers that have been degraded by microorganisms over hundreds of millions of years [2]. Therefore, plastic in the environment can persist for hundreds of years, accumulating over time and spreading over long distances.

Nevertheless, even in case of recalcitrant plastic polymers some biodegradation does occur. The process of biodegradation begins with the secretion of extracellular enzymes by microorganisms. The enzymes adhere to the polymer surface and begin to break down the polymer chains into shorter or smaller molecules such as oligomers, dimers and monomers [9]. Smaller molecules are water soluble and can pass through membranes to be utilized as carbon and energy sources [10]. Under aerobic conditions, the end products are carbon dioxide and water, and under anaerobic conditions methane can also be formed [11]. In recent years, numerous studies have shown that various microorganisms, both prokaryotes and eukaryotes, can depolymerise and in some cases (partially) mineralise plastics. Fungi are the most efficient eukaryotes that can do this [2]. To name just a few examples, biodegradation has been reported for *Aspergillus flavus* on polyethylene (PE) [12] and several *Cladosporium* spp. and other species on polyurethane (PU) [13,14]. Some fungal cutinases and lipases have been used to achieve hydrolysis of polyethylene terephthalate (PET) [15]. *Coniochaeta hoffmannii* and *Pleurostoma richardsiae* have been shown to partially degrade polypropylene (PP) [16]. Fungi are among the most efficient natural decomposers, producing a wide range of extracellular enzymes and degrading some of the most recalcitrant natural compounds, from lignin to xenobiotics. Their key advantage over bacteria is their ability to thrive in stressful environments with low nutrient availability, low pH or low availability of water [17]. In the case of filamentous fungi, their hyphae can penetrate solid materials and accelerate decomposition. Fungi can also secrete hydrophobic proteins, hydrophobins, which facilitate interaction with the hydrophobic surface of plastics [18].

Microbial plastic degraders can be used for composting plastic polymers into inorganic compounds, primarily CO_2_ and H_2_O. Alternatively, microorganisms can be a source of enzymes that can depolymerise plastics into oligomers and monomers, which in turn can be re-used to make new polymers, as part of a truly circular economy of plastics [2,19]. Despite some initial successes, both approaches are still at the research stage. To improve the efficiency and cost-effectiveness of such processes, new and better plastic degraders need to be found.

The experimental approaches and methods used in the search for plastic-degrading microbes vary considerably from study to study and are often suboptimal [2]. This leads to variations in experimental procedures, data analysis, and reporting, making comparison and reproducibility of results between different studies difficult. For example, many studies use commercially available plastics as test substrates and track potential degradation by substrate weight loss. However, commercial plastics are not pure polymers but are mixed with potentially numerous additives used for bulking and colour, added as stabilisers to protect against heat, light or oxygen, as flame retardants, as plasticizers to improve flexibility and for various other purposes [20]. These compounds can inhibit microbial growth or, more worryingly in the case of biodegradation studies, provide a better substrate for the microbes than the plastic polymer itself. This can lead to false positives, where the growth of microorganisms and the decrease in plastic weight are interpreted as the degradation of a plastic polymer, when in fact microorganisms only degrade additives [2,21]. The lack of standardised screening methods is reflected in the relatively small number of microorganisms that have been tested so far.

In this study, we systematically tested 146 strains of fungi exposed to five pure plastic polymers and measured their metabolism with gas chromatography to detect the production of carbon dioxide. We focused on fungal isolates from two environments: car repair workshops (71 strains), an environment rich in long-chain hydrocarbons, and hypersaline water (75 strains), as most mismanaged plastic waste ends up in the oceans and on the seashores, at a rate of 8 million tonnes per year or more [22,23]. After this initial screening, the effects of the nine most promising candidates on various plastics were investigated using scanning electron microscopy (SEM), Fourier transform infrared spectroscopy (FTIR), and Raman spectroscopy.

## 2 Materials and methods

### 2.1 Strains and growth conditions

Strains for this study (Table 1 and Table 2) were obtained from the Culture Collection Ex of the Infrastructural Centre Mycosmo (Department of Biology, Biotechnical Faculty, University of Ljubljana, Slovenia). Cultures were maintained on malt extract agar (MEA) consisting of 2 % malt extract (Biolife, Italy), 0.1 % peptone (Condalab, Spain), 2 % glucose (Kemika, Croatia), and 2 % agar (Formedium, United Kingdom) in deionized water.

**Table 1:** List of strains isolated from habitats contaminated with long-chain or aromatic hydrocarbons used in this study.

Culture Collection Strain Number	Genus	Isolation Habitat and Location
EXF-6835	*Saccharomyces cerevisiae*	Petrol station; petrol-contaminated concrete floor surface; Slovenia, Grosuplje
EXF-6836	*Acremonium sclerotigenum*	Car repair workshop; oil/petrol contaminated surface; Slovenia, Škofja Loka
EXF-6837	*Graphium basitruncatum*	Car repair workshop; oil/petrol contaminated surface; Slovenia, Škofja Loka
EXF-6838	*Metschnikowia fructicola*	Car repair workshop; oil/petrol contaminated surface; Slovenia, Škofja Loka
EXF-6839	*Cryptococcus albidus*	Car repair workshop; oil/petrol contaminated surface; Slovenia, Škofja Loka
EXF-6840	*Peterozyma toletana*	Petrol station; petrol-contaminated concrete floor surface; Slovenia, Grosuplje
EXF-6841	*Cystobasidium lysinophilum*	Car repair workshop; car parking surface; Slovenia, Škofja Loka
EXF-6842	*Cystobasidium slooffiae*	Petrol station; petrol-contaminated concrete floor surface; Slovenia, Slovenj Gradec
EXF-6843	*Rhodotorula diobovata*	Petrol station; petrol-contaminated concrete floor surface; Slovenia, Slovenj Gradec
EXF-6844	*Meyerozyma guilliermondii*	Petrol station; petrol-contaminated concrete floor surface; Slovenia, Grosuplje
EXF-6845	*Cystobasidium slooffiae*	Petrol station; petrol-contaminated concrete floor surface; Slovenia, Grosuplje
EXF-6846	*Cryptococcus uniguttulatus*	Petrol station; petrol-contaminated concrete floor surface; Slovenia, Grosuplje
EXF-6848	*Wickerhamomyces anomalus*	Car repair workshop; oil/petrol contaminated surface; Slovenia, Tolmin
EXF-6849	*Alternaria* sp.	Petrol station; petrol-contaminated concrete floor surface; Slovenia, Celje
EXF-6850	*Phialophora* sp.	Petrol station; handle of the diesel fuel nozzle; Slovenia, Celje
EXF-6852	*Fusarium oxysporum*	Car repair workshop floor; Slovenia, Tolmin
EXF-6853	*Naganishia albida*	Petrol station; petrol-contaminated concrete floor surface; Slovenia, Grosuplje
EXF-6858	*Chaetomium globosum*	Car repair workshop; oil/petrol contaminated surface; Slovenia, Celje
EXF-6859	*Penicillium* sp.	Petrol station; petrol-contaminated concrete floor surface; Slovenia, Krško
EXF-6863	*Exophiala dermatitidis*	Rubber seal at the opening of the car petrol reservoir; Slovenia, Ivančna Gorica
EXF-6866	*Pichia fermentans*	Petrol station; petrol-contaminated concrete floor surface; Slovenia, Ljubljana
EXF-6869	*Candida saitoana*	Car repair workshop floor; Slovenia, Ivančna Gorica
EXF-6874	*Cystobasidium lysinophilum*	Car repair workshop floor; Slovenia, Ivančna Gorica
EXF-6875	*Trichosporon lactis*	Car repair workshop floor; Slovenia, Ivančna Gorica
EXF-6882	*Cystobasidium* sp.	Petrol station; Slovenia
EXF-6883	*Cystobasidium slooffiae*	Petrol station; Slovenia
EXF-6983	*Candida davisiana*	Petrol station; petrol-contaminated concrete floor surface; Slovenia, Ivančna Gorica
EXF-6984	*Hanseniaspora uvarum*	Petrol station; petrol-contaminated concrete floor surface; Slovenia, Ivančna Gorica
EXF-6985	*Exophiala dermatitidis*	Petrol station; petrol-contaminated concrete floor surface; Slovenia, Ivančna Gorica
EXF-6989	*Cystobasidium slooffiae*	Rubber seal at the opening of the car petrol reservoir; Slovenia, Ivančna Gorica
EXF-6993	*Cystobasidium lysinophilum*	Petrol station; handle of the petrol fuel nozzle; Slovenia, Rakek
EXF-7040	*Exophiala xenobiotica*	Petrol station; handle of the petrol fuel nozzle; Slovenia, Grosuplje
EXF-10600	*Epicoccum nigrum*	Car fuel reservoir, diesel; Slovenia
EXF-10601	*Cladosporium* sp.	Car fuel reservoir, diesel; Slovenia
EXF-10603	*Trichoderma* sp.	Car fuel reservoir, diesel; Slovenia
EXF-10604	*Aspergillus* sp.	Car fuel reservoir, petrol; Slovenia
EXF-10605	*Cladosporium* sp.	Car fuel reservoir, petrol; Slovenia
EXF-10605	*Cladosporium* sp.	Car fuel reservoir, petrol; Slovenia
EXF-10625	*Exophiala xenobiotica*	Car fuel reservoir; Slovenia
EXF-10626	*Rhodotorula* sp.	Car fuel reservoir; Slovenia
EXF-10627	*Cryptococcus* sp.	Car fuel reservoir; Slovenia
EXF-10628	*Metschnikowia fructicola*	Car fuel reservoir; Slovenia
EXF-10629	*Aureobasidium pullulans*	Car fuel reservoir; Slovenia
EXF-10630	*Rhodotorula* sp.	Car fuel reservoir, diesel; Slovenia
EXF-10631	*Cryptococcus taibaiensis*	Car fuel reservoir, diesel; Slovenia
EXF-10632	*Aureobasidium pullulans*	Car fuel reservoir, diesel; Slovenia
EXF-13286	*Coniochaeta hoffmannii*	Car repair workshop, mixture of oil and water; Slovenia, Ljubljana
EXF-13287	*Coniochaeta hoffmannii*	Car fuel reservoir, diesel; Slovenia, Ljubljana
EXF-13290	*Exophiala phaeomuriformis*	Car repair workshop, mixture of oil and water; Slovenia, Ljubljana
EXF-13291	*Cladosporium* sp.	Car fuel reservoir, diesel; Slovenia, Ljubljana
EXF-13292	*Dipodascus* sp.	Car repair workshop, mixture of oil and water; Slovenia, Ljubljana
EXF-13293	*Meyerozyma guilliermondii*	Car repair workshop, mixture of oil and water; Slovenia, Ljubljana
EXF-13296	*Meyerozyma caribbica*	Car repair workshop, mixture of oil and water; Slovenia, Ljubljana
EXF-13298	*Rhodotorula diobovata*	Car repair workshop, mixture of oil and water; Slovenia, Ljubljana
EXF-13299	*Exophiala dermatitidis*	Car repair workshop, mixture of oil and water; Slovenia, Ljubljana
EXF-13300	*Aureobasidium melanogenum*	Car fuel reservoir, diesel; Slovenia, Ljubljana
EXF-13304	*Aureobasidium pullulans*	Car fuel reservoir, diesel; Slovenia, Ljubljana
EXF-13305	*Exophiala bergeri*	Car fuel reservoir, diesel; Slovenia, Ljubljana
EXF-13306	*Candida boidinii*	Car repair workshop, mixture of oil and water; Slovenia, Ljubljana
EXF-13308	*Pleurostoma richardsiae*	Car repair workshop, mixture of oil and water; Slovenia, Ljubljana
EXF-13309	*Acremonium sclerotigenum*	Car repair workshop, mixture of oil and water; Slovenia, Ljubljana
EXF-13310	*Exophiala dermatitidis*	Car repair workshop, mixture of oil and water; Slovenia, Ljubljana
EXF-13311	*Geotrichum* sp.	Car repair workshop, mixture of oil and water; Slovenia, Ljubljana
EXF-13315	*Rhodotorula* sp.	Car fuel reservoir, diesel; Slovenia, Ljubljana
EXF-13317	*Penicillium* sp.	Car repair workshop, mixture of oil and water; Slovenia, Ljubljana
EXF-13500	*Rhodotorula dairenensis*	Water shaft in a car repair shop; Slovenia, Ljubljana
EXF-13501	*Cystobasidium lysinophilum*	Car fuel reservoir, diesel; Slovenia, Ljubljana
EXF-13502	*Cladosporium* sp.	Water pump in a car repair workshop; Slovenia, Ljubljana
EXF-13503	*Cladosporium cladosporioides*	Car fuel reservoir, diesel; Slovenia, Ljubljana
EXF-13504	*Meyerozyma guilliermondii*	Water shaft in a car repair workshop; Slovenia, Ljubljana
EXF-13505	*Candida boidinii*	Water shaft in a car repair workshop; Slovenia, Ljubljana
EXF-13507	*Alternaria* sp.	Car fuel reservoir, diesel; Slovenia, Ljubljana
EXF-13508	*Cladosporium allicinum*	Car fuel reservoir, diesel; Slovenia, Ljubljana
EXF-14537	*Penicillium citrinum*	Creosote oil (0.1 %) contaminant; Slovenia

**Table 2:** List of strains isolated from hypersaline habitats used in this study.

Culture Collection Strain Number	Genus	Isolation Habitat and Location
EXF-82	*Aspergillus fumigatus*	Active salterns; Slovenia, Sečovlje
EXF-150	*Aureobasidium pullulans*	Active salterns; Slovenia, Sečovlje
EXF-172	*Neophaeotheca triangularis*	Active salterns; Slovenia, Sečovlje
EXF-174	*Alternaria alternata*	Saltpans; Slovenia, Sečovlje
EXF-181	*Microascus brevicaulis*	Active salterns; Slovenia, Sečovlje
EXF-185	*Aspergillus pseudoglaucus*	Active salterns; Slovenia, Sečovlje
EXF-186	*Aspergillus silvaticus*	Active salterns; Slovenia, Sečovlje
EXF-191	*Aspergillus candidus*	Active salterns; Slovenia, Sečovlje
EXF-198	*Aspergillus restrictus*	Active salterns; Slovenia, Sečovlje
EXF-295	*Trimmatostroma salinum*	Active salterns; Slovenia, Sečovlje
EXF-310	*Aspergillus versicolor*	Active salterns; Slovenia, Sečovlje
EXF-322	*Cladosporium salinae*	Active salterns; Slovenia, Sečovlje
EXF-334	*Cladosporium spinulosum*	Active salterns; Slovenia, Sečovlje
EXF-343	*Cladosporium subinflatum*	Active salterns; Slovenia, Sečovlje
EXF-348	*Aspergillus filifera*	Active salterns; Slovenia, Sečovlje
EXF-349	*Aspergillus stella-maris*	Active salterns; Slovenia, Sečovlje
EXF-374	*Alternaria botrytis*	Saltpans; Slovenia, Sečovlje
EXF-389	*Cladosporium allicinum*	Active salterns; Slovenia, Sečovlje
EXF-397	*Cladosporium fusiforme*	Active salterns; Slovenia, Sečovlje
EXF-401	*Aspergillus tubingensis*	Active salterns; Slovenia, Sečovlje
EXF-402	*Aspergillus europaeus*	Active salterns; Slovenia, Sečovlje
EXF-405	*Aspergillus sydowii*	Active salterns; Slovenia, Sečovlje
EXF-407	*Aspergillus chevalieri*	Active salterns; Slovenia, Sečovlje
EXF-411	*Aspergillus westerdijkiae*	Active salterns; Slovenia, Sečovlje
EXF-418	*Aspergillus amstelodami*	Active salterns; Slovenia, Sečovlje
EXF-430	*Aspergillus* sp.	Active salterns; Slovenia, Sečovlje
EXF-441	*Aspergillus ruber*	Active salterns; Slovenia, Sečovlje
EXF-462	*Cladosporium subtilissimum*	Active salterns; Slovenia, Sečovlje
EXF-466	*Cladosporium velox*	Active salterns; Slovenia, Sečovlje
EXF-512	*Rhodotorula sphaerocarpa*	Active salterns; Slovenia, Sečovlje
EXF-513	*Rhodotorula babjevae*	Active salterns; Slovenia, Sečovlje
EXF-514	*Meyerozyma guilliermondii*	Active salterns; Slovenia, Sečovlje
EXF-517	*Candida parapsilosis*	Active salterns; Slovenia, Sečovlje
EXF-670	*Aspergillus alliaceus*	Saltpans; Slovenia, Sečovlje
EXF-770	*Penicillium polonicum*	Active salterns; Slovenia, Sečovlje
EXF-785	*Pseudoscopulariopsis schumacheri*	Active salterns; Slovenia, Sečovlje
EXF-795	*Penicillium fundyense*	Active salterns; Slovenia, Sečovlje
EXF-810	*Aspergillus sclerotiorum*	Active salterns; Slovenia, Sečovlje
EXF-951	*Wallemia muriae*	Active salterns; Slovenia, Sečovlje
EXF-994	*Wallemia ichthyophaga*	Saltpans; Slovenia, Sečovlje
EXF-2250	*Meira* sp.	Saltpans; Slovenia, Sečovlje
EXF-2277	*Sarocladium strictum*	Saltpans; Slovenia, Sečovlje
EXF-2287	*Cladosporium macrocarpum*	Saltpans; Slovenia, Sečovlje
EXF-2294	*Rhizopus* sp.	Saltpans; Slovenia, Sečovlje
EXF-2317	*Alternaria tenuissima*	Saltpans; Slovenia, Sečovlje
EXF-2331	*Alternaria* sp.	Saltpans; Slovenia, Sečovlje
EXF-2340	*Alternaria arborescens*	Saltpans; Slovenia, Sečovlje
EXF-4289	*Aspergillus ustus*	Active salterns; Slovenia, Sečovlje
EXF-4341	*Aspergillus glaucus*	Active salterns; Slovenia, Sečovlje
EXF-4498	*Apiospora montagnei*	Active salterns; Slovenia, Sečovlje
EXF-4670	*Acremonium* sp.	Active salterns; Slovenia, Sečovlje
EXF-4676	*Beauveria bassiana*	Active salterns; Slovenia, Sečovlje
EXF-4680	*Verticillium* sp.	Active salterns; Slovenia, Sečovlje
EXF-5141	*Leptobacillium chinense*	Pre-crystalization pond brine, first layer; Slovenia, Sečovlje
EXF-5146	*Aspergillus welwitschiae*	Pre-crystalization pond brine, first layer; Slovenia, Sečovlje
EXF-5161	*Rhinocladiella similis*	Pond in inactive salterns, second layer; Slovenia, Sečovlje
EXF-5411	*Aspergillus terreus*	Saltern mud (fango); Slovenia, Sečovlje
EXF-5431	*Aspergillus pseudoglaucus*	Saltern mud (fango); Slovenia, Sečovlje
EXF-5442	*Humicola fuscoatra*	Saltern mud (fango); Slovenia, Sečovlje
EXF-5447	*Emericellopsis pallida*	Saltern mud (fango); Slovenia, Sečovlje
EXF-5464	*Aspergillus proliferans*	Saltern mud (fango); Slovenia, Sečovlje
EXF-5473	*Emericellopsis* sp.	Saltern mud (fango); Slovenia, Sečovlje
EXF-5477	*Aspergillus chevalieri*	Saltern mud (fango); Slovenia, Sečovlje
EXF-5480	*Chaetomium* sp.	Saltern mud (fango); Slovenia, Sečovlje
EXF-6033	*Candida tropicalis*	Young petola from saltern pond S10; Slovenia, Sečovlje
EXF-6904	*Apiospora arundinis*	Salty wood; Slovenia, Sečovlje
EXF-6905	*Apiospora sphaerosperma*	Unknown; Slovenia, Sečovlje
EXF-6910	*Bjerkandera adusta*	Saltern brine; Slovenia, Sečovlje
EXF-7729	*Papiliotrema laurentii*	*Arthrocnemum* sp. plant in salterns; Slovenia, Sečovlje
EXF-8985	*Exophiala dermatitidis*	Saltern mud (fango); Slovenia, Sečovlje
EXF-8986	*Aureobasidium melanogenum*	Saltern mud (fango); Slovenia, Sečovlje
EXF-10803	*Preussia persica*	Saltern mud (fango); Slovenia, Sečovlje
EXF-10810	*Aspergillus* sp.	Bittern (magnesium rich saltern brine after halite precipitation); Slovenia, Sečovlje
EXF-10827	*Penicillium brevicompactum*	Saltern brine; Slovenia, Sečovlje
EXF-10866	*Aspergillus domesticus*	Bittern (magnesium rich saltern brine after halite precipitation); Slovenia, Sečovlje
EXF-10879	*Penicillium bialowiezense*	Bittern (magnesium rich saltern brine after halite precipitation); Slovenia, Sečovlje
EXF-10920	*Cladosporium pulvericola*	Bittern (magnesium rich saltern brine after halite precipitation); Slovenia, Sečovlje
EXF-10927	*Verrucocladosporium dirinae*	Bittern (magnesium rich saltern brine after halite precipitation); Slovenia, Sečovlje
EXF-13753	*Rhodotorula* sp.	Saltern brine; Slovenia, Sečovlje
EXF-13754	*Vishniacozyma victoriae*	Saltern brine; Slovenia, Sečovlje
EXF-13776	*Penicillium sizovae*	Saltern brine; Slovenia, Sečovlje
EXF-13782	*Penicillium terrigenum*	Saltern brine; Slovenia, Sečovlje
EXF-13783	*Penicillium jacksonii*	Saltern brine; Slovenia, Sečovlje
EXF-13813	*Vishniacozyma heimaeyensis*	Saltern brine; Slovenia, Sečovlje
EXF-14064	*Aspergillus ruber*	Saltern brine; Slovenia, Sečovlje

### 2.2 Preparation of gas-tight serum bottles

Five different types of plastic, namely low-density polyethylene (LDPE), polyamide (PA), polyethylene terephthalate (PET), polypropylene (PP), and polyurethane (PU) (all GoodFellow, United Kingdom; Table 3), in the form of films (LDPE, PA, PET, PP) and foam (PU), were cut into 1 cm^2^ pieces and then sterilized by incubation in 96 % ethanol for 30 min [16]. After sterilization, the plastics were removed from the ethanol and dried at 37 °C until all residual ethanol evaporated, at least one week after there was no visible ethanol left. Plastics were then added to autoclave-sterilized gas-tight serum bottles containing 25 mL of mineral medium (MM) with no carbon source: 0.6 % NaNO_3_ (Kemika, Croatia), 0.15 % KH_2_PO_4_ (Sigma-Aldrich, USA), 0.05 % KCl (Merck, Germany), 0.05 % MgSO_4_ (Carlo Elba, Italy), 0.00002 % FeSO_4_ × 7H_2_O (Sigma-Aldrich, USA), 2 mL Trace Elements stock solution (modified according to Vishniac and Santer [24]) in double-distilled (MiliQ) water. For the first screening, one piece of each of the five plastics was placed in the bottle. For the second screening, in which the degradation of the individual types of plastic was evaluated, five pieces of a single type of plastic were placed in the bottle.

**Table 3:** Types of plastics used in this study and their properties.

Type of plastics	Form	Thickness	Color
Low-Density Polyethylene (LDPE)	Film	0.025 mm	Transparent
Polyamide – Nylon 6,6 (PA 6,6)	Film	0.017 mm	Transparent
Polyethylene terephthalate (PET)	Film	0.013 mm	Transparent
Polypropylene (PP)	Film	0.012 mm	Transparent
Polyurethane (PU)	Foam	10 mm	Grey

A subset of strains from hypersaline environments has also been tested in MM media with high concentrations of NaCl (from 10 to 25 % (w/v)), as listed in Supplementary Table S1. *Wallemia ichthyophaga*, which does not grow in media without added salt, was tested at 5 % and 25 % NaCl (w/v).

### 2.3 Inoculation of gas-tight serum bottles

In the case of yeasts, cell suspensions were prepared in saline solution (0.9 % NaCl (w/v) in MiliQ water). The optical density of the cell suspensions at a wavelength of 600 nm was measured using a spectrophotometer (Shimadzu UV-1800, Switzerland) and was adjusted to OD 1.0. Sporogenous cultures were inoculated as spore suspension with OD_600_ 0.5. Five hundred microliters of the cell or spore suspensions were pipetted into gas-tight serum bottles containing MM medium.

For filamentous strains where cell suspensions could not be prepared, the gas-tight serum bottles containing MM medium were inoculated with a mycelial plug (4 mm diameter) from the edge of an actively growing colony. The methods of inoculation used for each strain are listed in Supplementary Table S2.

**Fig. 1:**
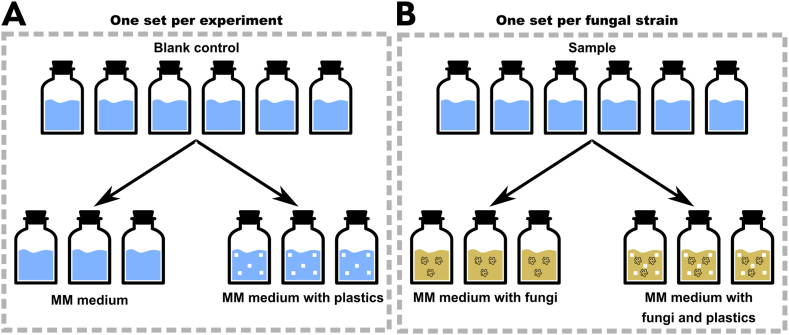
Schematic representation of inoculation of gas-tight serum bottles for measuring the production of CO_2_ during co-incubation of fungi and plastic polymers. **A**. Control experiments without fungal inoculation. **B**. Serum bottles inoculated with fungi with or without plastic polymers as the only source of carbon in the media.

We used six bottled as blank controls – three with MM medium only and three with MM medium and plastic, all without inoculation (Fig. 1A). For each fungal strain, we prepared six gas-tight serum bottles – three without plastic pieces and three with plastic pieces (Fig. 1B).

All gas-tight serum bottles were incubated at 25 °C for 4 weeks.

### 2.4 Gas chromatography

Gas chromatography was used to determine the production of carbon dioxide (CO_2_) in gas-tight serum bottles after four weeks of incubation. We used a gas chromatograph (Hewlett Packard 5890A Gas Chromatograph, USA) equipped with a thermal conductivity detector (TCD) and a 180 cm column with a diameter of 1/8˝ and filling Porapak R mesh 100/120 (Millipore Corporation, Massachusetts, USA). Separation and detection were performed under the following chromatographic conditions: oven temperature 50 °C, injector temperature 100 °C, TCD detector temperature 100 °C, carrier gas helium (180 mL/min), integrator HP3392A [25].

The amount of CO_2_ produced in the blank controls was subtracted from the amounts obtained in the samples. Then we calculated the average and the standard deviation (SD) for the three biological replicates and compared the amount of CO_2_ in the bottles with plastic to the amount of CO_2_ in the bottles without plastic. For samples in which the average share of CO_2_ in bottles with plastics was larger than the average CO_2_ share without plastics, the significance of the difference was checked with a one-tailed Student t-test in R [26].

After screening the strains on mixed plastics, we selected the nine best CO_2_ producers from car repair workshops for a screening with individual plastics using the same procedure as described above, but with only a single type of plastic per bottle. After this screening, we selected the fungus-plastic combinations with the highest CO_2_ production and analyzed them further using scanning electron microscopy (SEM), FTIR, and Raman spectroscopy, as described below.

### 2.5 Inoculation of plastic films on solid medium

Sterile plastic film pieces of different types of plastic were placed on MM medium without a carbon source. In the case of yeasts, strains were inoculated with a 10-μL inoculation loop in the centre of the plastic film pieces. In the case of filamentous fungi, strains were inoculated with 4-mm mycelial plugs in the centre of the plastic film pieces. Three replicates were prepared for each type of plastic. For controls, the plastic film pieces were placed on MM medium without inoculation. The plates were incubated at 24 °C for two months and then stored at 4 °C until further analysis.

### 2.6 Scanning electron microscopy (SEM)

Scanning electron microscopy (SEM) was used for a general assessment of fungal growth on different plastic films. Plastic films were collected from the growth media and placed directly on the SEM holder without prior drying or coating of the sample since environmental SEM analysis (ESEM, Quanta 650, Thermo Fisher Scientific, Waltham, Massachusetts, USA) allows low vacuum imaging.

### 2.7 Fourier-transform infrared spectroscopy (FTIR)

After the incubation, the film samples (three replicates per treatment) were washed with distilled water and then analyzed using attenuated total reflectance Fourier-transform infrared spectroscopy (FTIR-ATR) was applied using an Agilent Cary 630 spectrometer, equipped with a single reflection diamond crystal. FTIR-ATR spectra were acquired in the mid-infrared (MIR) region (4000–400 cm^−1^) with a resolution of 4 cm^−1^ and performing 200 scans. A background was obtained before each measurement. Films of each plastic (three replicates) without treatment were used as controls. For LDPE, PA, PET, and PP, twenty-five measurements were performed on each replicate of the plastic films arranged on a grid of 1.5 × 1 cm approx. For PU, given the size and shape of the foam samples (i.e., prisms), we only performed 10 measurements along the longest axis.

Standardized spectra, second derivative spectra, average spectra and standard deviation spectra were obtained using the R package ‘andurinha’ [26,27]. The same package was used to identify the wavenumbers of the relevant bands (based on the second derivative spectra) and obtain standardized absorbance values for them. The assignment of bands was based on existing literature [[28], [29], [30], [31], [32], [33], [34], [35]].

Instead of using individual absorbances to trace changes in plastic composition, we performed principal components analysis (PCA) on relevant MIR bands (standardized absorbance values) for each plastic. In this way, bands that covary are allocated to the same component that constitutes a spectral signal. Before the PCA, the variability of each plastic replicate (9 for PA and 6 for LDPE, PET, PP and PU each; 735 spectra) was assessed based on the standard deviation spectrum, which provides a measure of spatial homogeneity/heterogeneity of the film composition (as reflected by the MIR spectral properties). As described below, the control film replicates showed almost identical standard deviation spectra with very low values, while those of incubated films had similar spectra as control samples but with larger values. For the sake of this research, the PCA was performed using the measurements taken in two (three in the case of PA) replicates per plastic: one of the control films and another of the films incubated with fungi (the one that showed the largest standard deviation spectrum). ANOVA analysis was also applied to the principal components’ scores to assess significant differences in MIR signals between control and incubated films.

The relative decrease in absorbance (RAD), a proxy for changes in plastic composition (i.e., degradation), was calculated for the bands with large loadings on the principal components that showed significant differences between control and incubated films, according to Equation (1).

Equation 1 $$RAD\;\left(\%\right)=\frac{average\;absorbance\;after\;incubation\;-\;average\;absorbance\;before\;incubation}{average\;absorbance\;before\;incubation}\times 100$$

### 2.8 Raman spectroscopy

In addition to FTIR, Raman spectroscopy was used to similarly evaluate the possible chemical changes in plastic films after fungal colonization. Using a confocal Raman spectrometer (Integra Spectra I, NT-MDT Co., Russia), a blue wavelength laser with 488 nm was focused on the plastic sample surface at positions with and without fungi, in Raman shift range from 200 to 3000 cm^−1^. Spectra were acquired upon 10 s of exposure with 5 times accumulation. Three spectra were taken to get the average at each position. For every spectrum, baseline absorbance was subtracted, and treated samples were normalized to untreated controls to determine relative changes in different bond vibrations.

## 3 Results

**Fig. 2:**
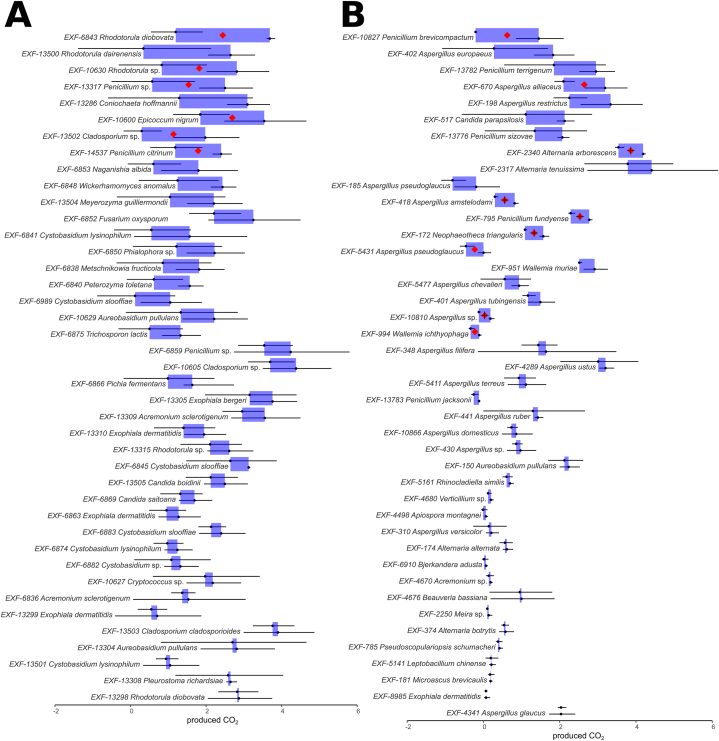
Production of CO_2_ by fungi incubated in the presence of plastics as the only carbon source (right border of blue rectangles with the corresponding standard deviation as error bars), compared to the production of CO_2_ in the absence of plastic (left border of blue rectangles with the corresponding standard deviation as error bars). The value of CO_2_ is expressed as a percent proportion in the total gas phase of the test bottle. A mixture of five plastic polymers was used: low-density polyethylene (LDPE), polyamide (PA), polyethylene terephthalate (PET), polypropylene (PP), and polyurethane (PU). Only strains with increased CO_2_ production in the presence of plastic are shown. Statistically significant changes (one-tailed Student t-test) upon exposure to plastics are marked with red diamonds (p < 0.05) and “+” signs (p < 0.01). **A**. Fungal strains isolated from car repair workshops. **B**. Fungal strains isolated from hypersaline environments. (For interpretation of the references to colour in this figure legend, the reader is referred to the Web version of this article.)

Of the 146 strains tested for the CO_2_ production when exposed to a mixture of plastic polymers (compared to the same medium without the plastic polymers), 41 strains isolated from car repair workshops and 42 strains isolated from salterns (of 71 or 75 tested, respectively) on average released more CO_2_ when exposed to plastic polymers (Supplementary Fig. S1). For car repair workshop strains (Fig. 2A), this difference was significant for six strains (*t*-test, p < 0.05). For saltern strains (Fig. 2B), the difference was significant for nine (*t*-test, p < 0.05) strains and highly significant for five of these (*t*-test, p < 0.01). Differences between biological replicates were relatively large, as visible from large standard deviation lines on Fig. 2. In a large number of samples the amount of released CO_2_ decreased in samples incubated with plastic polymers – these samples are shown on Supplementary Fig. S1.

Twelve strains released significantly higher amounts of CO_2_ when exposed to plastics in media supplemented with high concentrations of NaCl (Supplementary Fig. S1). Most of these belonged to the genus *Aspergillus* (7 strains), two strains to *Penicillium*, and one strain to each of the genera *Aureobasidium*, *Cladosporium*, and *Petromyces*. Some of these strains also showed an increased CO_2_ production in the medium without added salt (Supplementary Fig. S1).

**Fig. 3:**
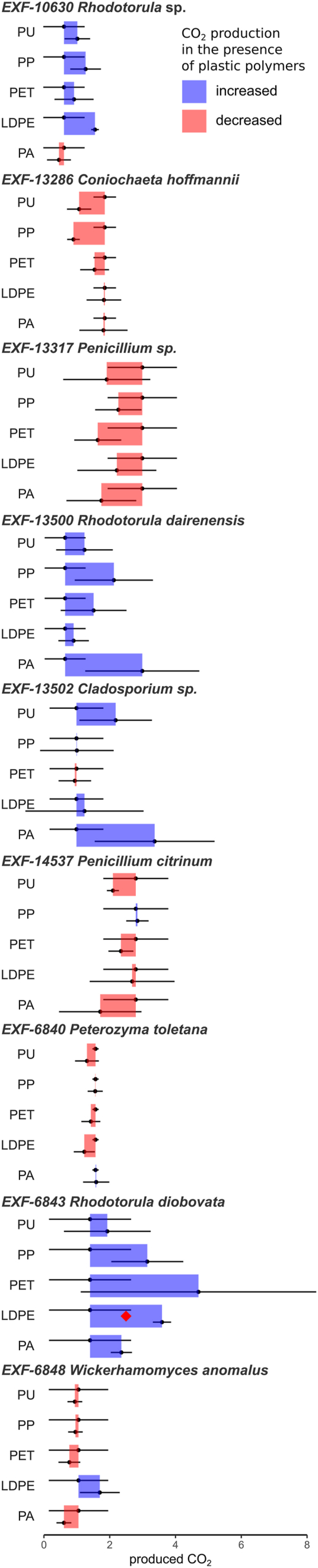
Production of CO_2_ by fungi incubated in the presence of polyurethane (PU), polypropylene (PP), polyethylene terephthalate (PET), low-density polyethylene (LDPE), and polyamide (PA) as the only carbon source, compared to the production of CO_2_ in the absence of plastic. Left and right borders of coloured rectangles correspond to the amount of produced CO_2_ in each condition. Blue rectangles represent an increase in CO_2_ production in the presence of plastic, and red rectangles represent a decrease, both with corresponding standard deviations as error bars. The value of CO_2_ is expressed as a percent proportion in the total gas phase of the test bottle. Statistically significant increases (one-tailed Student t-test) in CO_2_ in the presence of plastics are marked with red diamonds (p < 0.05). (For interpretation of the references to colour in this figure legend, the reader is referred to the Web version of this article.)

Nine of the strains from car repair workshops that were among the best CO_2_ producers in media with mixed plastics were selected for a more detailed screening. The strains were inoculated to identical media as in the original screening, but in this case, supplemented with each of the five plastic polymers individually. For four of these strains, the plastic-stimulated release of CO_2_ was not confirmed for any of the polymers (Fig. 3). *Wickerhamomyces anomalus* (EXF-6848) produced more CO_2_ on PET, while *Rhodotorula* sp. (EXF-10630), *Rhodotorula dairenensis* (EXF-13500), *Cladosporium* sp. (EXF-13502), and *Rhodotorula diobovata* (EXF-6843) produced more CO_2_ on most plastics. However, the difference was only significant in the case of *R. diobovata* (EXF-6843) on LDPE (*t*-test, p < 0.05).

Based on these results, the following fungi and polymers were selected for further analyses: *W. anomalus* (EXF-6848) on PET, *Rhodotorula* sp. (EXF-10630) on LDPE, *R. dairenensis* (EXF-13500) on PA and PP, *Cladosporium* sp. (EXF-13502) on PA, and *R. diobovata* (EXF-6843) on PU.

**Fig. 4:**
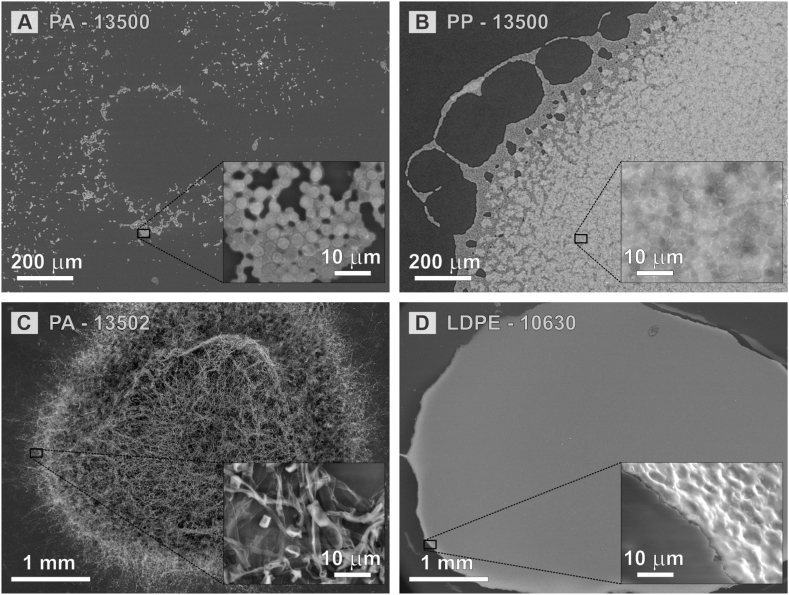
Scanning electron microscopy images of fungi growing on plastic polymer foils incubated on a minimal medium without a source of carbon. **A.***Rhodotorula dairenensis* EXF-13500 spread poorly across the PA surface and **B.** extensively on the PP surface. **C.***Cladosporium* sp. EXF-13502 overgrew PA surface and **D.***Rhodotorula* sp. EXF-10630 grew well on LDPE.

Fungal growth on different plastic films was visualized using SEM (Fig. 4). Different types of plastic samples were inspected after 2 months of incubation. Analysis showed clear differences in the growth between the more and less colonised plastic samples. For example, as shown in Fig. 4, *R. dairenensis* EXF-13500 did not cover much of the PA foil, with only small islands of cells found isolated on some parts of the plastic surface (Fig. 4A). On the other hand, when the same strain was incubated on PP foil, extensive growth was observed (Fig. 4B). Extensive overgrowth was also observed for *Cladosporium* sp. EXF-13502 on PA foil (Fig. 4C). An even denser colonization over a large portion of the surface was observed in the case of *Rhodotorula* sp. EXF-10630 on LDPE foil. In this case the boundary between the colonized and uncolonized regions of plastic was much clearer than in other samples (Fig. 4D).

**Fig. 5:**
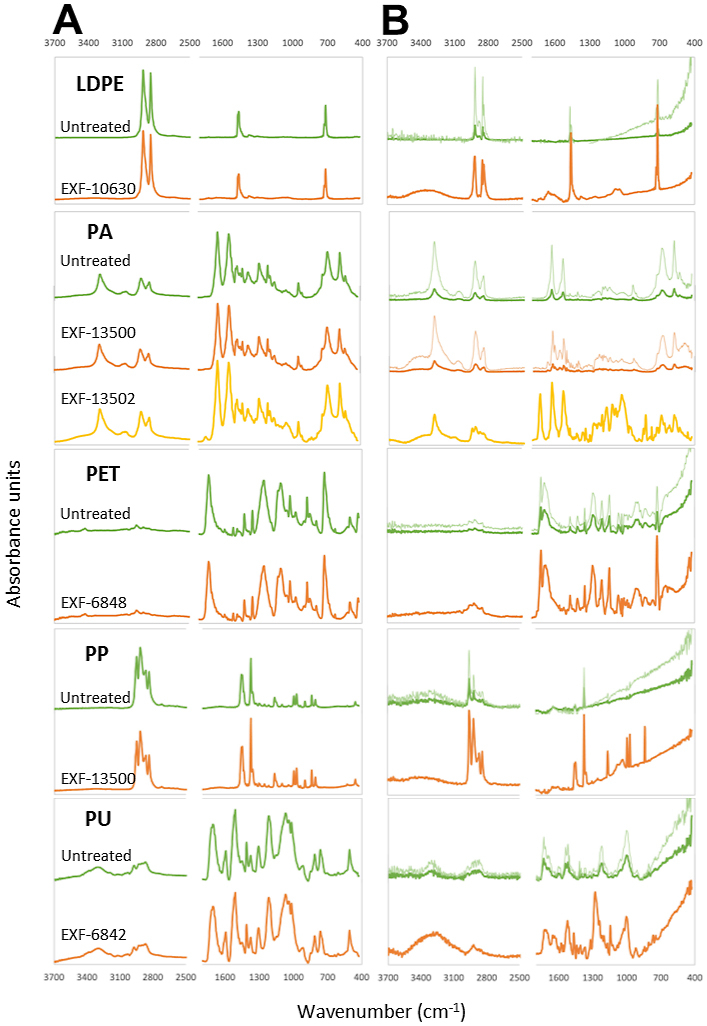
Average (**A**) and standard deviation (**B**) spectra of the analyzed plastics (control films and films co-incubated with various fungal strains: *Rhodotorula diobovata* (EXF-6843), *Wickerhamomyces anomalus* (EXF-6848), *Rhodotorula* sp. (EXF-10630), *Rhodotorula dairenensis* (EXF-13500), and *Cladosporium* sp. (EXF-13502)). The thick lines in the standard deviation spectrum correspond to the actual values, while the thin line is the spectrum multiplied by a constant value so variations can be more easily observed. The plastics polymers are low-density polyethylene (LDPE), and polyamide (PA), polyethylene terephthalate (PET), polypropylene (PP), polyurethane (PU).

The FTIR-ATR spectra of the control and incubated film samples are represented in Fig. 5 as the average (Fig. 5A) and standard deviation (Fig. 5B) spectra. All characteristic absorption bands and the corresponding bond vibrations can be found in Supplementary Table S3. The low-density polyethylene (LDPE) spectrum shows the characteristic bands corresponding to –CH_2_- stretching (st, 2915 and 2840 cm^−1^), in-plane bending (bd-ip, 1463 and 1377 cm^−1^), and C–H rocking (rk, 718 cm^−1^). The polyamide 6.6 (PA) spectrum shows bands corresponding to amide I (C=O st at 1631 cm^−1^), amide II (-NH bd-ip and C–N st at 1534 cm^−1^, and an overtone at 3066 cm^−1^), amide III (C–N st and –NH bd-ip at 1275 cm^−1^), amide IV (N–H out of plane bending, bd-oop, at 688 cm^−1^), amide V (N–H bd-oop at 729 cm^−1^), N–H st (3297 cm^−1^), C–H and –CH_2_ stretching and bending (2932, 2857, 1474, 1461, 1439, 1416, 1370, 1198, and 1180 cm^−1^), C=O stretching and bending (1731, 936, 578 cm^−1^), as well as chain deformations (533 cm^−1^). The polyethylene terephthalate (PET) spectrum shows characteristic bands of C–H stretching and bending (1409, 1349, 1018, 969, 870, 846, and 725 cm^−1^), C=O stretching (1715 cm^−1^), O–C stretching (1124 and 1098 cm^−1^), and C–C stretching (1243 cm^−1^). The PP spectrum is characterized by absorbances corresponding to –CH_2_ and –CH_3_ stretching and bending vibrations (2917, 2950, 2868, 2838, 1459, 1437, 1375, 1359, 1167, 997, 973, 900, and 841 cm^−1^), and the C–C stretching of the polymer backbone (809 cm^−1^). And the polyurethane (PU) spectrum shows absorbances of CH vibrations (2900-2800, 1375, 1308, and 1016 cm^−1^), C–C vibrations (1411, 1040, and 813 cm^−1^), C–N and N–H vibrations (1593 and 1511 cm^−1^), C=O (1705 cm^−1^) and C–O (1072 cm^−1^) vibrations, and O–C vibration (1217 cm^−1^). All absorption bands and the corresponding bond vibrations can be found in Supplementary Table S3.

The average spectra of the controls and films incubated with fungi are essentially the same, but the standard deviation spectra provided some interesting additional information. For all plastics, the spectrum of the control films showed low to very low values when compared to those of the incubated films (Fig. 5). The spectrum of film PA incubated with *R. dairenensis* EXF-13500 that showed values comparable to those of the control is an exception. In the incubated films, some regions/bands with overall low absorbance presented moderate to large variability, as the 1200-1000 cm^−1^ region in PA incubated with *Cladosporium* sp. EXF-13502, the 1090-1040 cm^−1^ region and 3400 cm^−1^ in PP incubated with *R. dairenensis* EXF-13500; while, in some cases, regions/bands with background absorbances in the control film showed moderate to large variability in the incubated film, as 3350, 1650, and 1090-1040 cm^−1^ in LDPE (incubated with *Rhodotorula* sp. EXF-10630), 1730 cm^−1^ in PA (incubated with *Cladosporium* sp. EXF-13502), and 3300 cm^−1^ in PU (incubated with *R. diobovata* EXF-6843) (Fig. 5).

The results of the PCA are synthesized in Supplementary Table S3. For each plastic, only components with at least one band with a loading ≥0.5 (or ≤ −0.5) were considered: two for LDPE and PP, three for PET and four for PA and PU. These components explained between 90 % and 94 % of the total MIR variance of the datasets used in the analysis. Communalities (i.e., sum of the squares of the loadings of a variable in the extracted components) were ≥0.80 for almost bands (Supplementary Table S3). Only one band in PA (936 cm^−1^), another in PU (1375 cm^−1^) and two in PET (1504 and 434 cm^−1^) were below 0.8, but well above 0.5. Thus, the extracted components can be considered as highly representative of the MIR spectral properties of the plastics.

**Fig. 6:**
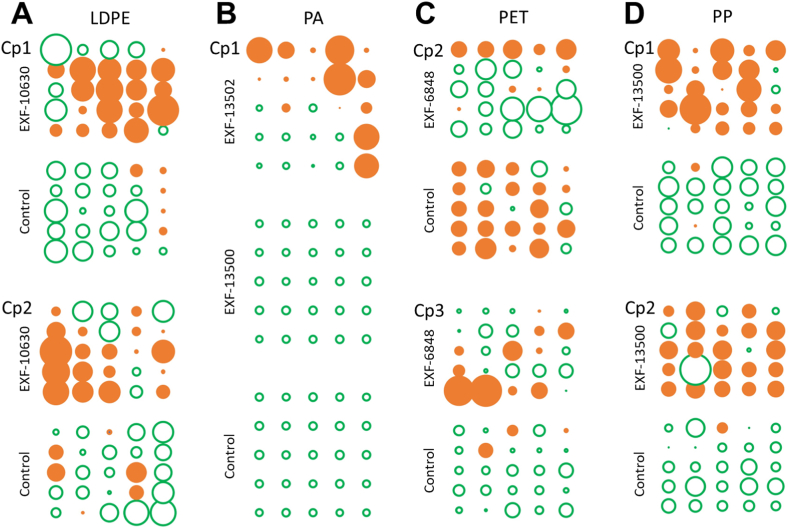
Scores of the extracted principal components that showed significant differences between the control films and the films co-incubated with various fungal strains: *Wickerhamomyces anomalus* (EXF-6848), *Rhodotorula* sp. (EXF-10630), *Rhodotorula dairenensis* (EXF-13500), and *Cladosporium* sp. (EXF-13502). Green circles correspond to negative scores and orange circles correspond to positive scores. The size of the circle indicates the actual score value. The plastics polymers are **A**. low-density polyethylene (LDPE), and **B**. polyamide (PA), **C**. polyethylene terephthalate (PET), **D**. polypropylene (PP). (For interpretation of the references to colour in this figure legend, the reader is referred to the Web version of this article.)

The ANOVA test showed significant differences between the control films and the incubated films (Supplementary Table S4) for the first component (Cp1) for PA, the first two components (Cp1 and Cp2) for LDPE and PP, the second and third (Cp2 and Cp3) for PET, and none for PU. The spatial variation of the scores of these significant components is represented in Fig. 6.

In LDPE, Cp1 showed negative or slightly positive scores in the control film and mostly large positive scores in the film incubated with *Rhodotorula* sp. EXF-10630 (Fig. 6A). In the incubated films C–H stretching vibrations decreased (negative loadings, Supplementary Table S3) while C–H bending vibrations increased (positive loadings). A similar response was found for Cp2, with mostly negative scores in the control film and positive in the incubated film (Fig. 6A). This second component accounts for a secondary variation at 1463 cm^−1^ (C–H asymmetric bending in plane) and implies that absorbance increased in the incubated film. While score values tend to be more homogeneous in the control film, the spatial distribution of scores is more heterogenous and almost symmetrical in the film incubated with fungi, indicating that some regions of the plastic were more affected than others.

For PA only Cp1 showed significant differences (Fig. 6B–Supplementary Table S4). The scores are negative, low, and highly homogeneous in the control film. This indicates higher absorbances of the amide vibrations (negative loadings, Supplementary Table S3). For the incubated films, *R. dairenensis* EXF-13500 showed the same spatial pattern of the control film while *Cladosporium* sp. EXF-13502 showed positive score values on the fringes of the film (Fig. 6B). This suggests that *Cladosporium* sp. EXF-13502 produced a decrease in absorbance of the amide bonds and a relative increase in C–H stretching and bending (2857 and 1180 cm^−1^) but *R. dairenensis* EXF-13500 seems to have had no effect on the film composition.

In PET, Cp2 had positive scores values for the control film, indicating larger relative absorbance of the carbonyl (C=O) and O–C stretching and C–H bending of the aromatic ring. These absorbances were decreased in the incubated film (*W*. *anomalus* EXF-6848), while the C=C stretching of the aromatic ring (1504 cm^−1^) was relatively increased. Cp3 scores were negative and quite homogeneous in the control film, which indicates that the control film had lower relative absorbance in the C–H wagging of the aromatic ring than the incubated film. The spatial distribution of the scores (Fig. 6C) shows that the two components reflect changes in different areas of the incubated film.

Components Cp1 and Cp2 showed significant differences between the control film and the incubated film (*R. dairenensis* EXF-13500) for PP (Fig. 6D). In both cases the spatial distribution of the scores is quite homogenous, with negative values in the control film and dominantly positive in the incubated film. This indicates that absorbances of the methyl and methylene stretching (bands in the 3000-2900 cm^−1^) were higher in the control film than in the incubated film, while methyl and methylene bending vibrations and C–C stretching (including the polymer backbone) were higher in the later.

**Fig. 7:**
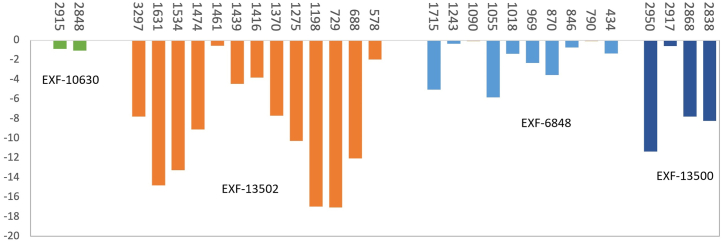
The relative decrease in absorbance (RAD) for bands with high loadings on the principal components that showed significant differences between the control films and the incubated films: *Wickerhamomyces anomalus* (EXF-6848), *Rhodotorula* sp. (EXF-10630), *Rhodotorula dairenensis* (EXF-13500), and *Cladosporium* sp. (EXF-13502). Only bands showing a decrease are shown. The plastics polymers are low-density polyethylene (LDPE), and polyamide (PA), polyethylene terephthalate (PET), polypropylene (PP).

To obtain a measure of the overall changes induced by the incubation with the fungi, we calculated the relative decrease in absorbance (RAD, formula 1 in material and methods) for the bands related to the components that showed significant differences between the control films and the incubated films. The extent of the decrease in absorbance varied within and between the incubated films (Fig. 7). The largest decreases (more than 10 %) were found for PA with *Cladosporium* sp. EXF-13502 and PP with *R*. *dairenensis* EXF-13500; overall moderate to low decreases (1–7%) were observed in PET (*W*. *anomalus* EXF-6848) and low decreases (1 %) in LDPE (*Rhodotorula* sp. EXF-10630).

**Fig. 8:**
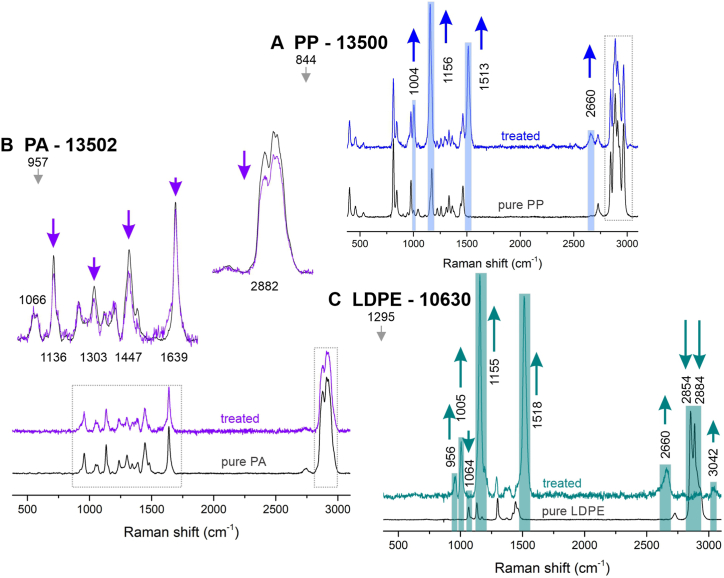
Raman spectroscopy reveals structural changes of plastics due to overgrowth of (A) *Rhodotorula dairenensis* EXF-13500 on PP, (B) *Cladosporium* sp. EXF-13502 on PA and (C) *Rhodotorula* sp. EXF-10630 on LDPE foil. The plastics polymers are low-density polyethylene (LDPE), and polyamide (PA), polyethylene terephthalate (PET), polypropylene (PP).

Confocal Raman spectroscopy successfully distinguished between the naked plastic surface and the surface covered with fungi, but also resolved the polymer structure under the thin film of fungal cells. Several of the investigated fungus-polymer combinations exhibited notable changes in spectra (Fig. 8): PP with *R. dairenensis* EXF-13500, PA with *Cladosporium* sp. EXF-13502 and LDPE with *Rhodotorula* sp. EXF-10630. Raw spectra recorded over the inhibited area greatly increase the noise-to-peak ratio for various reasons. Normalization alters the relative changes of the peak intensities belonging to different functional groups.

Changes were observed in PP with *R. dairenensis* EXF-13500, by normalizing intensities to the CH_2_ rocking at 844 cm^−1^ (Fig. 8A). A noticeable decrease in the relative intensities at 402 cm^−1^ is associated with CH_2_ vibration and CH bending. Peaks at 812 cm^−1^ corresponding to CH_2_ rocking and stretching of C–C and C–CH_3_ and the ones positioned at 2843–2967 cm^−1^, corresponding to population of methyl groups and CH_2_ symmetric and asymmetric stretching, also decreased. A significant increase was observed at 1004 cm^−1^, which is associated with CH_3_ rocking and at 1156 cm^−1^ C–C stretching with CH bending. Along the skeletal polymer structure new peaks appeared at 1513 and 2660 cm^−1^. For sample PA with *Cladosporium* sp. EXF-13502 (Fig. 8B), with normalizing intensities to the CCO stretching at 957 cm^−1^, a smaller decrease in the vibrations for C–C skeletal stretching at 1066 and 1136 cm^−1^, at 1303 cm^−1^ twisting NH, 1447 cm^−1^ bending CH_2_, 1639 cm^−1^ C=O stretching and at 2882 cm^−1^ CH stretching was observed. In contrast to the previous sample no new peaks were found in the vicinity or under areas colonized by the fungus. LDPE with *Rhodotorula* sp. EXF-10630 was normalized to 1295 cm^−1^ CH_2_ twisting and the intensity at 1064 cm^−1^ corresponding to C–C stretching almost completely disappear in the region colonized with fungi (Fig. 8C). Peaks at 1132 cm^−1^ C–C stretching and at 1173 cm^−1^ CH_2_ rocking are coupled into a large peak positioned at 1155 cm^−1^, which appears at the same position where C–C backbone resonance is located in PP (Fig. 8A). 1374 cm^−1^ CH_3_ wagging does not grow much over the sigma value while similarly as in the sample PP with *R. dairenensis* EXF-13500 a slightly shifted new peak appears at 1518 cm^−1^, which overshadows various CH_2_ banding modes represented at 1420, 1443 and 1462 cm^−1^. Along with this, new peaks appear at 956, 1005 2660, and 3042 cm^−1^.

## 4 Discussion

Biodegradation of plastics can be tracked with a variety of methods. While no widely accepted consensus has formed yet about the standard methodology, some approaches are becoming increasingly common. Among them are gravimetric measurements of the plastic substrate and the increase in biomass of the test organism, scanning electron microscopy (SEM), Fourier-transform infrared spectroscopy (FTIR), or the use of isotopically labelled plastics [2,36]. In this study we used an initial screening of 146 fungal strains by tracking their CO_2_ production during growth in the presence of plastic polymers, followed by SEM, FTIR and Raman spectroscopy for best-performing candidates.

Fungi are natural decomposers and have several advantages over bacteria when it comes to biodegradation: growth in extreme environments, production of hydrophobins and, in the case of filamentous fungi, mechanical degradation of plastic. Their growth is relatively slow compared to bacteria and they are generally less adaptable [37]. However, fungi can produce various enzymes that may be of interest for the degradation of plastics. Respirometry can provide quantitative information on plastic degradation [36], but has one important limitation: it can only detect mineralisation of the polymer (or part of it). Microorganisms that are only capable of depolymerisation, but not of further utilization of oligomers or monomers, cannot be detected with such an approach. Such microorganisms might still be relevant for plastic degradation, either as part of microbial consortia for composting of plastics or as a source of enzymes for plastic depolymerisation and re-use of the monomers in a circular economy of plastics [38].

Despite this limitation, our results show that respirometry can identify at least some microorganisms with potential for plastic degradation. Surprisingly, we also detected several cases of decreased CO_2_ production upon exposure to plastic polymers. The reason for this could simply be a relatively high variability between biological replicates, stemming from small differences in the initial inoculum (which can be difficult in the case of filamentous fungi, but should not present a problem with yeasts) or the length of incubation amplifying minute differences in the seal between individual incubation flasks. Alternatively, the presence of plastic polymers may be detrimental to the growth of some fungi. This would be difficult to explain with the published literature. Organic carbon can leach from plastics, but in a lake ecosystem, it was found to stimulate microbial growth rather than inhibit it [39]. Furthermore, we used pure plastic polymers with no additives. Such plastics can still release residual monomeric and oligomeric content [40], but most of this would have happened during the ethanol pre-treatment of the plastics and not during incubation.

Due to all these reasons, the potential for plastic degradation identified by respirometry must be investigated by further tests. While the requirement for this additional confirmation increases the complexity of the screening approach, it also provides additional insight into the degradation capacity of individual microorganisms. Furthermore, additional tests are also required by other screening methods described in the literature, from growth tests to gravimetry, each with their own set of shortcomings, as described above. If the ultimate goal is the use of pure enzymes, screening and confirmation must be followed by the identification of these enzymes, their optimisation and production – steps requiring substantial efforts extending beyond the scope of initial screening.

As expected, each plastic had a characteristic MIR (FTIR-ATR) spectrum (Fig. 5), as found in previous investigations that applied the same technique [[28], [29], [30], [31], [32], [33], [34], [35]]. The studied plastics were easily identified using FTIR-ATR. Both the control plastic films and the plastic films co-incubated with fungi shared the same overall spectrum. But the standard deviation spectra showed that the presence of fungi resulted in a large increase in standard deviation (i.e., variability), with few exceptions (e.g., PA incubated with *Rhodotorula dairenensis* EXF-13500) (Fig. 5). Most transformations induced by the presence of fungi occurred in the characteristic bands of the plastics, but some bands had larger variability than others and even background regions in the control films showed moderate to large variability in the films exposed to fungi, indicating the formation of new bonds (e.g., carbonyl in PA incubated with *Cladosporium* sp. EXF-13502). Thus, increased superficial heterogeneity is a good marker of fungal enzymatic attack. Different species of fungi have different enzymatic profiles, which are also reflected in the type of plastic they can break down and the type of bonds they can act on [41].

PCA enabled us to synthesize the MIR data into a few spectral signals (2–4 components). ANOVA on the components scores indicated that almost all plastics were significantly affected by fungi in ways that were specific for individual fungal strains: C–H stretching was reduced in LDPE (*Rhodotorula* sp. EXF-10630), amide bonds in PA (*Cladosporium* sp. EXF-13502), carbonyl, O–C bond, and aromatic C–H in PET (*Wickerhamomyces anomalus* EXF-6848), and methyl and methylene bonds in PP (*R. dairenensis* EXF-13500). PA was not affected by *R. dairenensis* EXF-13500 and no changes were detected in PU after exposure to *Rhodotorula diobovata* EXF-6843. Importantly, while PA exposed to *R. dairenensis* EXF-13500 showed the same variability (i.e., standard deviation spectrum) as the control film, in PU the variability of the spectra increased after the exposure to the fungus (Fig. 5). While this may indicate an effect of the fungus on PU, the result may also be due to the lower number of measurements done in the PU foams (10 instead of 25), because of the shape of the samples. It is also interesting to note that *R. dairenensis* EXF-13500 did not affect PA but it produced extensive changes in PP, also reflected in the growth of this strain visible on SEM images in Fig. 4A and B. This underlines the need for the identification of microorganisms best suited for specific polymers, conditions and desired outputs [2], rather than searching for a universal plastic degrader.

Despite the significant changes indicated by ANOVA, the relative absorbance decrease (RAD) showed that plastics and bands were differentially affected (Fig. 7). *Cladosporium* sp. EXF-13502 produced the largest RAD values in PA, and *R*. *dairenensis* EXF-13500 in PP (except for CH_2_ stretching, 2917 cm^−1^), while values were moderate to low in PET (*W*. *anomalus* EXF-6848) and low in LDPE (*Rhodotorula* sp. EXF-10630).

Confocal Raman spectroscopy results confirmed many of the FTIR conclusions (Fig. 8). Spectra obtained on the control plastic were characteristic for PP, PA and LDPE [[42], [43], [44], [45]]. In the regions colonized by fungi, the peak intensities were reduced, as expected due to the diminished polymer structure [42,43,46]. Also, the noise signal was larger due to the excitation in fungi, but not showing any clear resonance. Upon normalization to a single peak, the relative differences between the intensity of the peaks for non-colonized and colonized plastic foils were noticeable. While less pronounced in *Cladosporium* sp. EXF-13502 on PA (Fig. 8B), more noticeable changes were observed for *R. dairenensis* EXF-13500 on PP and *Rhodotorula* sp. EXF-10630 on LDPE (Fig. 8A and C).

Changes in the peak intensities under the fungi partially depended on the density of fungal growth. In the center of the colony, where the amount of biomass is high, Raman spectra were very difficult to resolve. Moving towards the edge of this same region, the underlying plastic started to exhibit a characteristic vibrational fingerprint, with intensities increasingly matching the spectra obtained from the non-colonized areas. Raman signal is generated in micrometer-sized illuminated regions and the observed heterogeneity within the same plastic foil based on the local growth of the fungus also explains some observations of the FTIR measurements. FTIR covers much larger (up to millimeter-sized) areas, which can substantially differ in fungal colonization, resulting in a large standard deviation in measurements taken on a particular plastic sample. As expected, the variation in measurement of a pristine polymer film was much smaller.

As detected by FTIR, Raman spectroscopy also detected new peaks in the signal after the fungal treatment of plastics. This was particularly noticeable for peaks at 1155 cm^−1^, 1515 cm^−1^ and 2660 cm^−1^ (in PP and LDPE), originating from small regions with dense fungal growth.

Identification of three *Rhodotorula* spp. basidiomycetous yeasts as potential plastic degraders was perhaps surprising since yeasts are often thought to be worse enzyme producers than filamentous fungi. However, various species from the genus *Rhodotorula* are known for their enzyme production and nutritional versatility, making them attractive candidates for use in biotechnology, biocontrol and bioremediation [[47], [48], [49]]. Some species of the genus have already been linked to the degradation of plastic polymers or additives. A strain of *Rhodotorula rubra* was shown to partially degrade several plasticizers [50]. A strain of an unidentified species of the genus degraded polyester–polyether urethane [13,51]. The genus was also enriched in mealworms fed with PE [52].

Species from the genus *Cladosporum* have also been implicated in plastic degradation before. *Cladosporium cladosporioides* can carry out initial degradation of low-density PE [53]. *Cladosporium halotolerans* partially degrades high-density PE [54], and – like several other species of the genus – also polyester PU [55]. The genus is large and diverse and has considerable potential for plastic degradation, as also supported by the results of this study.

The final aspect of this study that warrants further research is the biodegradation of plastics at increased salinity. Many strains producing significantly more CO_2_ in the presence of plastic and salt belonged to the genus *Aspergillus*, a large genus with many species implicated in plastic degradation [2,56]. A large part of improperly discarded plastics reaches the oceans, at a rate of 8 million tonnes per year [22], already amounting to over 300 million tonnes [23]. Degradation of plastics at high salinity is thus a field of particular importance. As indicated by our preliminary results, biodegradation in such conditions may be possible and warrants further research.

This methodology is the first step in the search for fungal plastic degraders and forms the basis for further studies of the complex dynamics of plastic degradation using molecular analysis.

## 5 Conclusions

Despite its inability to detect depolymerisation of plastic polymers without further utilization, respirometric detection of CO_2_ production is a suitable method for screening the fungal potential for plastics biodegradation. Like other methods used for this purpose, this approach is relatively work-intense, which limits its scalability, but it can be streamlined by testing several plastic polymers in the same sample bottle and only testing them individually for a small subset of best-performing fungal strains. For these, respirometry should be followed by methods such as SEM, FTIR or Raman spectroscopy before claims of plastic polymer degradation can be made. If degradation is confirmed, the identification of enzymes involved in the process is one of the steps towards their potential use for real-life plastic biodegradation.

## Data availability

All data of this study is included in the article or supplementary materials.

## CRediT authorship contribution statement

**Anja Černoša:** Writing – review & editing, Writing – original draft, Visualization, Methodology, Investigation, Conceptualization. **Antonio Martínez Cortizas:** Writing – review & editing, Writing – original draft, Visualization, Methodology, Investigation, Formal analysis. **Mohamed Traoré:** Writing – review & editing, Investigation, Formal analysis. **Matejka Podlogar:** Writing – review & editing, Visualization, Methodology, Investigation. **Tjaša Danevčič:** Writing – review & editing, Resources, Investigation. **Nina Gunde-Cimerman:** Writing – review & editing, Funding acquisition. **Cene Gostinčar:** Writing – review & editing, Writing – original draft, Visualization, Supervision, Project administration, Methodology, Investigation, Funding acquisition, Formal analysis, Conceptualization.

## Declaration of competing interest

The authors declare that they have no known competing financial interests or personal relationships that could have appeared to influence the work reported in this paper.

## Figures and Tables

**figs1:**