# Supplementary material for: A screening method for plastic-degrading fungi
Source: Heliyon. 2024 May 11;10(10):e31130. doi: 10.1016/j.heliyon.2024.e31130 (PMC11128935; doi:10.1016/j.heliyon.2024.e31130)
Supplement: Multimedia component 1 [file mmc1.pdf]

Supplementary Table S1. Strains from hypersaline environments tested for increased production of CO_2 when growing in the presence of plastic polymers and at high concentrations of NaCl.

EXF number	Species	The tested concentration of NaCl (% (w/v))
EXF-82	*Aspergillus fumigatus*	10
EXF-150	*Aureobasidium pullulans*	15
EXF-172	*Neophaeotheca triangularis*	20
EXF-174	*Alternaria alternata*	20
EXF-185	*Aspergillus pseudoglaucus*	20
EXF-186	*Aspergillus silvaticus*	15
EXF-191	*Aspergillus candidus*	20
EXF-198	*Aspergillus restrictus*	20
EXF-295	*Trimmatostroma salinum*	20
EXF-310	*Aspergillus versicolor*	20
EXF-322	*Cladosporium salinae*	15
EXF-334	*Cladosporium spinulosum*	15
EXF-343	*Cladosporium subinflatum*	15
EXF-348	*Emericella filifera*	20
EXF-349	*Emericella stella-maris*	20
EXF-389	*Cladosporium allicinum*	10
EXF-397	*Cladosporium fusiforme*	15
EXF-401	*Aspergillus tubingensis*	20
EXF-402	*Aspergillus europaeus*	20
EXF-405	*Aspergillus sydowii*	20
EXF-407	*Eurotium chevalieri*	20
EXF-411	*Aspergillus westerdijkiae*	20
EXF-418	*Aspergillus amstelodami*	20
EXF-430	*Aspergillus wentii*	20
EXF-441	*Eurotium rubrum*	15
EXF-462	*Cladosporium subtillissimum*	10
EXF-466	*Cladosporium velox*	20
EXF-512	*Rhodosporidium sphaerocarpa*	20
EXF-513	*Rhodosporidium babjevae*	10
EXF-517	*Candida parapsilosis*	15
EXF-670	*Aspergillus alliaceus*	20
EXF-770	*Penicillium polonicum*	20
EXF-795	*Penicillium fundyense*	20
EXF-810	*Aspergillus sclerotiorum*	15
EXF-951	*Wallemia muriae*	25
EXF-994	*Wallemia ichthyophaga*	25
EXF-2287	*Cladosporium macrocarpum*	15

EXF-2317	*Alternaria tenuissima*	15
EXF-2331	*Alternaria sp.*	10
EXF-2340	*Alternaria arborescens*	15
EXF-4289	*Aspergillus ustus*	15
EXF-4341	*Aspergillus glaucus*	15
EXF-5146	*Aspergillus welwitschiae*	15
EXF-5411	*Aspergillus terreus*	20
EXF-5431	*Aspergillus pseudoglaucus*	20
EXF-5447	*Emericellopsis pallida*	15
EXF-5464	*Aspergillus proliferans*	20
EXF-5477	*Aspergillus chevalieri*	20
EXF-6033	*Candida tropicalis*	10
EXF-8986	*Aureobasidium melanogenum*	15
EXF-10810	*Aspergillus proliferans/glaucus*	20
EXF-10827	*Penicillium brevicompactum*	20
EXF-10866	*Aspergillus domesticus*	20
EXF-10879	*Penicillium bialowiezense*	20
EXF-10920	*Cladosporium dominicanum*	20
EXF-13753	*Rhodotorula graminis*	10
EXF-13776	*Penicillium sizovae*	20
EXF-13782	*Penicillium terrigenum*	20
EXF-13783	*Penicillium jacksonii*	15
EXF-14064	*Aspergillus ruber*	20

Supplementary Table S2: List of strains used in this study and their inoculation method.

**Culture Collection Strain Number**	**Genus**	**Inoculation method**
EXF-6835	*Saccharomyces cerevisiae*	cell suspension
EXF-6836	*Acremonium sclerotigenum*	cell suspension
EXF-6837	*Graphium basitruncatum*	mycelial plug
EXF-6838	*Metschnikowia fructicola*	cell suspension
EXF-6839	*Cryptococcus albidus*	cell suspension
EXF-6840	*Peterozyma toletana*	cell suspension
EXF-6841	*Cystobasidium lysinophilum*	cell suspension
EXF-6842	*Cystobasidium slooffiae*	cell suspension
EXF-6843	*Rhodotorula diobovata*	cell suspension
EXF-6844	*Meyerozyma guilliermondii*	cell suspension
EXF-6845	*Cystobasidium slooffiae*	cell suspension
EXF-6846	*Cryptococcus uniguttulatus*	cell suspension
EXF-6848	*Wickerhamomyces anomalus*	cell suspension
EXF-6849	*Alternaria* sp.	mycelial plug
EXF-6850	*Phialophora* sp.	mycelial plug
EXF-6852	*Fusarium oxysporum*	mycelial plug
EXF-6853	*Naganishia albida*	cell suspension
EXF-6858	*Chaetomium globosum*	mycelial plug
EXF-6859	*Penicillium* sp.	mycelial plug
EXF-6863	*Exophiala dermatitidis*	cell suspension
EXF-6866	*Pichia fermentans*	cell suspension
EXF-6869	*Candida saitoana*	cell suspension
EXF-6874	*Cystobasidium lysinophilum*	cell suspension
EXF-6875	*Trichosporon lactis*	cell suspension
EXF-6882	*Cystobasidium* sp.	cell suspension
EXF-6883	*Cystobasidium slooffiae*	cell suspension
EXF-6983	*Candida davisiana*	cell suspension
EXF-6984	*Hanseniaspora uvarum*	cell suspension
EXF-6985	*Exophiala dermatitidis*	cell suspension
EXF-6989	*Cystobasidium slooffiae*	cell suspension
EXF-6993	*Cystobasidium lysinophilum*	cell suspension
EXF-7040	*Exophiala xenobiotica*	mycelial plug
EXF-10600	*Epicoccum nigrum*	mycelial plug
EXF-10601	*Cladosporium* sp.	mycelial plug
EXF-10603	*Trichoderma* sp.	mycelial plug
EXF-10604	*Aspergillus* sp.	mycelial plug
EXF-10605	*Cladosporium* sp.	mycelial plug
EXF-10605	*Cladosporium* sp.	cell suspension
EXF-10625	*Exophiala xenobiotica*	cell suspension

EXF-10626	*Rhodotorula* sp.	cell suspension
EXF-10627	*Cryptococcus* sp.	cell suspension
EXF-10628	*Metschnikowia fructicola*	cell suspension
EXF-10629	*Aureobasidium pullulans*	cell suspension
EXF-10630	*Rhodotorula* sp.	cell suspension
EXF-10631	*Cryptococcus taibaiensis*	cell suspension
EXF-10632	*Aureobasidium pullulans*	cell suspension
EXF-13286	*Coniochaeta hoffmannii*	mycelial plug
EXF-13287	*Coniochaeta hoffmannii*	mycelial plug
EXF-13290	*Exophiala phaeomuriformis*	cell suspension
EXF-13291	*Cladosporium* sp.	cell suspension
EXF-13292	*Dipodascus* sp.	cell suspension
EXF-13293	*Meyerozyma guilliermondii*	cell suspension
EXF-13296	*Meyerozyma caribbica*	cell suspension
EXF-13298	*Rhodotorula diobovata*	cell suspension
EXF-13299	*Exophiala dermatitidis*	cell suspension
EXF-13300	*Aureobasidium melanogenum*	cell suspension
EXF-13304	*Aureobasidium pullulans*	cell suspension
EXF-13305	*Exophiala bergeri*	cell suspension
EXF-13306	*Candida boidinii*	cell suspension
EXF-13308	*Pleurostoma richardsiae*	mycelial plug
EXF-13309	*Acremonium sclerotigenum*	mycelial plug
EXF-13310	*Exophiala dermatitidis*	cell suspension
EXF-13311	*Geotrichum* sp.	cell suspension
EXF-13315	*Rhodotorula* sp.	cell suspension
EXF-13317	*Penicillium* sp.	spore suspension
EXF-13500	*Rhodotorula dairenensis*	cell suspension
EXF-13501	*Cystobasidium lysinophilum*	cell suspension
EXF-13502	*Cladosporium* sp.	mycelial plug
EXF-13503	*Cladosporium cladosporioides*	mycelial plug
EXF-13504	*Meyerozyma guilliermondii*	cell suspension
EXF-13505	*Candida boidinii*	cell suspension
EXF-13507	*Alternaria* sp.	mycelial plug
EXF-13508	*Cladosporium allicinum*	mycelial plug
EXF-14537	*Penicillium citrinum*	spore suspension
EXF-82	*Aspergillus fumigatus*	spore suspension
EXF-150	*Aureobasidium pullulans*	cell suspension
EXF-172	*Neophaeotheca triangularis*	spore suspension
EXF-174	*Alternaria alternata*	spore suspension
EXF-181	*Microascus brevicaulis*	mycelial plug
EXF-185	*Aspergillus pseudoglaucus*	spore suspension
EXF-186	*Aspergillus silvaticus*	spore suspension
EXF-191	*Aspergillus candidus*	spore suspension

EXF-198	*Aspergillus restrictus*	spore suspension
EXF-295	*Trimmatostroma salinum*	cell suspension
EXF-310	*Aspergillus versicolor*	spore suspension
EXF-322	*Cladosporium salinae*	spore suspension
EXF-334	*Cladosporium spinulosum*	spore suspension
EXF-343	*Cladosporium subinflatum*	spore suspension
EXF-348	*Aspergillus filifera*	spore suspension
EXF-349	*Aspergillus stella-maris*	spore suspension
EXF-374	*Alternaria botrytis*	spore suspension
EXF-389	*Cladosporium allicinum*	spore suspension
EXF-397	*Cladosporium fusiforme*	spore suspension
EXF-401	*Aspergillus tubingensis*	spore suspension
EXF-402	*Aspergillus europaeus*	spore suspension
EXF-405	*Aspergillus sydowii*	spore suspension
EXF-407	*Aspergillus chevalieri*	mycelial plug
EXF-411	*Aspergillus westerdijkiae*	spore suspension
EXF-418	*Aspergillus amstelodami*	spore suspension
EXF-430	*Aspergillus* sp.	spore suspension
EXF-441	*Aspergillus ruber*	spore suspension
EXF-462	*Cladosporium subtilissimum*	spore suspension
EXF-466	*Cladosporium velox*	spore suspension
EXF-512	*Rhodotorula sphaerocarpa*	cell suspension
EXF-513	*Rhodotorula babjevae*	cell suspension
EXF-514	*Meyerozyma guilliermondii*	cell suspension
EXF-517	*Candida parapsilosis*	cell suspension
EXF-670	*Aspergillus alliaceus*	spore suspension
EXF-770	*Penicillium polonicum*	spore suspension
EXF-785	*Pseudoscopulariopsis schumacheri*	mycelial plug
EXF-795	*Penicillium fundyense*	spore suspension
EXF-810	*Aspergillus sclerotiorum*	spore suspension
EXF-951	*Wallemia muriae*	spore suspension
EXF-994	*Wallemia ichthyophaga*	spore suspension
EXF-2250	*Meira* sp.	cell suspension
EXF-2277	*Sarocladium strictum*	cell suspension
EXF-2287	*Cladosporium macrocarpum*	spore suspension
EXF-2294	*Rhizopus* sp.	spore suspension
EXF-2317	*Alternaria tenuissima*	spore suspension
EXF-2331	*Alternaria* sp.	mycelial plug
EXF-2340	*Alternaria arborescens*	spore suspension
EXF-4289	*Aspergillus ustus*	mycelial plug
EXF-4341	*Aspergillus glaucus*	mycelial plug
EXF-4498	*Apiospora montagnei*	spore suspension
EXF-4670	*Acremonium* sp.	mycelial plug

EXF-4676	*Beauveria bassiana*	spore suspension
EXF-4680	*Verticillium* sp.	mycelial plug
EXF-5141	*Leptobacillium chinense*	spore suspension
EXF-5146	*Aspergillus welwitschiae*	spore suspension
EXF-5161	*Rhinocladiella similis*	spore suspension
EXF-5411	*Aspergillus terreus*	spore suspension
EXF-5431	*Aspergillus pseudoglaucus*	spore suspension
EXF-5442	*Humicola fuscoatra*	spore suspension
EXF-5447	*Emericellopsis pallida*	spore suspension
EXF-5464	*Aspergillus proliferans*	spore suspension
EXF-5473	*Emericellopsis* sp.	spore suspension
EXF-5477	*Aspergillus chevalieri*	mycelial plug
EXF-5480	*Chaetomium* sp.	spore suspension
EXF-6033	*Candida tropicalis*	cell suspension
EXF-6904	*Apiospora arundinis*	mycelial plug
EXF-6905	*Apiospora sphaerosperma*	spore suspension
EXF-6910	*Bjerkandera adusta*	mycelial plug
EXF-7729	*Papiliotrema laurentii*	cell suspension
EXF-8985	*Exophiala dermatitidis*	cell suspension
EXF-8986	*Aureobasidium melanogenum*	cell suspension
EXF-10803	*Preussia persica*	mycelial plug
EXF-10810	*Aspergillus* sp.	spore suspension
EXF-10827	*Penicillium brevicompactum*	spore suspension
EXF-10866	*Aspergillus domesticus*	mycelial plug
EXF-10879	*Penicillium bialowiezense*	spore suspension
EXF-10920	*Cladosporium pulvericola*	spore suspension
EXF-10927	*Verrucocladosporium dirinae*	mycelial plug
EXF-13753	*Rhodotorula* sp.	cell suspension
EXF-13754	*Vishniacozyma victoriae*	cell suspension
EXF-13776	*Penicillium sizovae*	spore suspension
EXF-13782	*Penicillium terrigenum*	spore suspension
EXF-13783	*Penicillium jacksonii*	spore suspension
EXF-13813	*Vishniacozyma heimaeyensis*	cell suspension
EXF-14064	*Aspergillus ruber*	spore suspension

Supplementary Table S3. Principal components analysis based on FTIR measurements. Relevant wavenumbers for each plastic with the associated vibrations and loadings of the significant extracted components. Large loadings (>0.5 or <-0.5) are in bold. Com: communalities.

LDPE		Cp1	Cp2	Cp3	Cp4	Com
718	C-H (-CH_2-) bd rk	**0.93**	-0.02			0.87
1377	C-H bd sm bd ip	**0.88**	0.33			0.89
1463	C-H as bd ip	**0.83**	**0.52**			0.97
2848	C-H (-CH_2-) sm st	**-0.84**	0.49			0.94
2915	C-H (-CH_2-) as st	**-0.92**	0.33			0.96
PA						
1180	C-H bd oop	**0.71**	-0.27	**0.53**	-0.34	0.97
2857	-CH_2 sm st	**0.60**	**-0.56**	-0.37	0.08	0.82
1416	-CH- [-CH-(CO-)]	**-0.64**	**0.56**	-0.29	-0.42	0.99
3297	NH st	**-0.72**	-0.39	**-0.54**	0.07	0.97
688	Amide IV (N-H bd-oop)	**-0.80**	0.47	0.28	0.10	0.96
1198	C-H bd oop	**-0.84**	-0.39	0.30	0.18	0.99
1439	C-H as bd ip	**-0.87**	-0.18	0.16	-0.41	0.97
729	Amide V (N-H bd-oop)	**-0.92**	0.15	0.33	0.13	0.99
1275	Amide III (C-N st & -NH bs ip)	**-0.93**	-0.29	0.12	0.16	0.98
1631	Amide I (C=O st)	**-0.94**	-0.18	-0.24	0.10	0.98
1370	C-H sm bd ip	**-0.94**	-0.27	0.06	0.09	0.97
1534	Amide II (-NH bd ip and C-N st)	**-0.95**	-0.07	-0.25	0.06	0.97
1474	-CH- [-CH-(NH)-]	**-0.98**	-0.06	-0.12	-0.05	0.98
533	chain conformations	0.08	**0.95**	0.14	-0.01	0.92
578	C=O bd-oop	-0.13	**0.91**	0.25	-0.06	0.90
936	C-C=O st	0.35	**0.71**	0.05	0.30	0.72
1731	C=O free OH	0.47	**-0.72**	0.47	0.16	0.99
2932	-CH_2- as st	0.34	**-0.87**	-0.18	0.05	0.90
3066	Amide II overtone	0.33	0.25	**-0.83**	-0.23	0.92
1461	C-H as bd ip	-0.32	**-0.53**	0.33	**-0.58**	0.82
PET						
969	C-H bd ip aromatic ring	**0.95**	0.18	-0.10		0.95
846	C-H (-CH_2-) bd rk	**0.93**	-0.21	-0.13		0.92
790	C-H_3 bd rk	**0.80**	-0.46	-0.27		0.93
434		**0.74**	-0.39	-0.14		0.72
870	C-H oop aromatic ring	**0.72**	**0.57**	-0.30		0.93
1055	C-O st	**0.71**	**0.54**	-0.36		0.93
1243	C-C(O)-O st	**-0.71**	**0.68**	-0.02		0.97
1340	C-H sm bd ip	**-0.82**	-0.14	-0.49		0.94
1124	O-C st	**-0.91**	-0.06	-0.22		0.87
1409	C-H sm bd ip	**-0.92**	-0.33	-0.13		0.97
1715	C=O ester st	-0.17	**0.84**	-0.48		0.96
1098	O-C st	-0.29	**0.79**	0.49		0.94

1018	C-H bd ip aromatic ring	0.48	**0.79**	0.17		0.88
1504	C=C st aromatic ring	**-0.55**	**-0.58**	-0.10		0.65
505		**0.57**	**-0.75**	-0.01		0.88
725	C-H bd wg aromatic ring	0.21	-0.11	**0.95**		0.95
PP						
973	CH_3 bd rk & C-C st	**0.96**	-0.08			0.93
809	C-C st polymer backbone	**0.96**	0.06			0.92
1167	CH_3 bd rk & C-C st	**0.93**	-0.08			0.88
997	$-CH_2$ bd rk+wg	**0.92**	-0.19			0.88
900	CH_2 bd-rk	**0.91**	-0.10			0.84
841	CH_2 bd-rk	**0.88**	-0.32			0.87
458		**0.88**	-0.20			0.81
1359	C-H ($-CH_2-$) sm bd ip	**0.87**	0.48			0.99
1437	C-H ($-CH_3-$) as bd ip	**0.87**	0.44			0.94
1375	C-H ($-CH_3-$) sm bd ip	**0.77**	**0.63**			0.99
1459	C-H ($-CH_2-$) as bd ip	**0.76**	**0.65**			0.99
2838	$-CH_2$ sm st	**-0.93**	0.21			0.91
2868	$-CH_3$ sm st	**-0.94**	0.27			0.96
2950	$-CH_3$ as st	**-0.94**	0.05			0.90
2917	$-CH_2$ as st	**-0.60**	**0.76**			0.94
PU						
1308	C-H ring	**-0.91**	0.22	-0.02	-0.21	0.93
1411	C-C st	**-0.89**	0.32	-0.04	-0.16	0.92
1511	C-N ad N-H	**-0.86**	0.19	-0.34	0.22	0.94
1705	C=O st	**-0.85**	0.33	-0.13	0.33	0.95
1217	[O-C(O)-O] st	**-0.82**	-0.27	-0.47	-0.06	0.96
813	C-C st polymer backbone	**-0.74**	**-0.51**	-0.08	-0.13	0.83
766		**-0.70**	**-0.67**	0.08	-0.18	0.97
1072	C-O-C st	**0.59**	-0.26	**-0.57**	-0.26	0.81
1016	C-H bd ip aromatic ring	**0.84**	-0.23	-0.02	0.29	0.85
1040	C-C st polymer backbone	**0.97**	0.00	-0.09	-0.19	0.98
1593	C-N st and N-H bd	0.25	**0.73**	-0.07	**-0.50**	0.85
509		-0.19	**-0.84**	0.34	-0.16	0.89
1375	CH_3 sm bd	-0.47	0.29	**0.69**	-0.09	0.78

Supplementary Table S4. Summary statistics of the ANOVA test on the scores of the extracted principal components.

		Sum of Squares	df	Mean Square	F	sig			N	Average	Std deviation	Std error
LDPECp1	Between Groups	12.771	1	12.771	16.921	0.000		Untreated	25	-0.505	0.505	0.101
	Within Groups	36.229	48	0.755				10630	25	0.505	1.120	0.224
	Total	49.000	49									
LDPECp2	Between Groups	9.443	1	9.443	11.459	0.001		Control	25	-0.435	0.755	0.151
	Within Groups	39.557	48	0.824				10630	25	0.435	1.038	0.208
	Total	49.000	49									
PACp1	Between Groups	20.748	2	10.374	14.026	0.000		Control	25	-0.379	0.035	0.007
	Within Groups	53.252	72	0.740				13500	25	-0.365	0.035	0.007
	Total	74.000	74					13502	25	0.744	1.489	0.298
PACp2	Between Groups	0.584	2	0.292	0.286	0.752		Control	25	0.125	0.387	0.077
	Within Groups	73.416	72	1.020				13500	25	-0.055	0.354	0.071
	Total	74.000	74					13502	25	-0.069	1.669	0.334
PACp3	Between Groups	3.730	2	1.865	1.911	0.155		Control	25	0.301	0.628	0.126
	Within Groups	70.270	72	0.976				13500	25	-0.068	0.568	0.114
	Total	74.000	74					13502	25	-0.233	1.487	0.297
PACp4	Between Groups	0.865	2	0.432	0.426	0.655		Control	25	0.009	0.248	0.050
	Within Groups	73.135	72	1.016				13500	25	0.127	0.218	0.044
	Total	74.000	74					13502	25	-0.136	1.714	0.343
PETCp1	Between Groups	3.173	1	3.173	3.323	0.075		Control	25	0.252	0.869	0.174
	Within Groups	45.827	48	0.955					25	-0.252	1.075	0.215
	Total	49.000	49									
PETCp2	Between Groups	12.139	1	12.139	15.808	0.000		Control	25	0.493	0.595	0.119
	Within Groups	36.861	48	0.768					25	-0.493	1.087	0.217
	Total	49.000	49									
PETCp3	Between Groups	5.169	1	5.169	5.661	0.021		Control	25	-0.322	0.432	0.086
	Within Groups	43.831	48	0.913					25	0.322	1.280	0.256
	Total	49.000	49									
PPCp1	Between Groups	30.300	1	30.300	77.776	0.000		Control	25	-0.778	0.417	0.083
	Within Groups	18.700	48	0.390					25	0.778	0.778	0.156
	Total	49.000	49									
PPCp2	Between Groups	15.468	1	15.468	22.142	0.000		Control	25	-0.556	0.457	0.091
	Within Groups	33.532	48	0.699					25	0.556	1.090	0.218
	Total	49.000	49									
PUCp1	Between Groups	2.672	1.000	2.672	2.946	0.103			10	-0.366	0.461	0.146
	Within Groups	16.328	18.000	0.907					10	0.366	1.265	0.400
	Total	19.000	19.000									
PUCp2	Between Groups	0.962	1.000	0.962	0.960	0.340			10	0.219	0.844	0.267
	Within Groups	18.038	18.000	1.002					10	-0.219	1.137	0.359
	Total	19.000	19.000									
PUCp3	Between Groups	1.062	1.000	1.062	1.065	0.316			10	-0.230	1.121	0.354
	Within Groups	17.938	18.000	0.997					10	0.230	0.859	0.272
	Total	19.000	19.000									
PUCp4	Between Groups	2.563	1.000	2.563	2.806	0.111			10	0.358	0.841	0.266
	Within Groups	16.437	18.000	0.913					10	-0.358	1.058	0.335
	Total	19.000	19.000									
